# A multinational observational study identifying primary care patients at risk of overestimation of asthma control

**DOI:** 10.1038/s41533-019-0156-4

**Published:** 2019-12-05

**Authors:** Vicky Kritikos, David Price, Alberto Papi, Antonio Infantino, Bjorn Ställberg, Dermot Ryan, Federico Lavorini, Henry Chrystyn, John Haughney, Karin Lisspers, Kevin Gruffydd-Jones, Miguel Román Rodríguez, Svein Høegh Henrichsen, Thys van der Molen, Victoria Carter, Sinthia Bosnic-Anticevich

**Affiliations:** 1 Quality Use of Respiratory Medicines Group, Woolcock Institute of Medical Research, University of Sydney, Sydney, NSW Australia; 2 Department of Respiratory and Sleep Medicine, Royal Prince Alfred Hospital, Sydney, NSW Australia; 3 Optimum Patient Care, Cambridge, UK; 4 Observational and Pragmatic Research Institute, Southbank, Singapore; 5 Academic Primary Care, University of Aberdeen, Aberdeen, UK; 6 Respiratory Medicine, University of Ferrara, Ferrara, Italy; 7 Special Interest Respiratory Area, Italian Interdisciplinary Society for Primary Care, Bari, Italy; 8 Department of Public Health and Caring Sciences, Family Medicine and Preventive Medicine, Uppsala University, Uppsala, Sweden; 9 Allergy and Respiratory Research Group, Usher Institute of Population Health Sciences and Informatics, University of Edinburgh, Edinburgh, UK; 10 Department Experimental and Clinical Medicine, University of Florence, Firenze, Italy; 11 Inhalation Consultancy Ltd, Leeds, UK; 12 NHS Greater Glasgow & Clyde R&D, Glasgow, UK; 13 Box Surgery, Box, UK; 14 Primary Care Respiratory Research Unit, Instituto de Investigación Sanitaria de Baleares (IdISBa), Palma, Spain; 15 Department of Primary Health Care Services, Norwegian Directorate of Health, Oslo, Norway; 16 Department of Primary Care, University of Groningen, University Medical Centre Groningen, Groningen, Netherlands; 17 Sydney Medical School, Faculty of Medicine and Health, The University of Sydney, Sydney, NSW Australia; 18 Central Sydney Local Area Health District, Sydney, NSW Australia

**Keywords:** Patient education, Health services

## Abstract

Factors related to the discrepancy between patient-perceived and actual disease control remain unclear. Identifying patients at risk of overestimation of asthma control remains elusive. This study aimed to (i) investigate the relationship between patient-reported and actual level of asthma control (ii), compare the characteristics between patients who believe their asthma is well controlled that accurately report ‘well-controlled’ asthma with those that do not, and (iii) identify factors associated with inaccurately reported ‘well-controlled’ asthma. A historical, multinational, cross-sectional study using data from the iHARP (initiative Helping Asthma in Real-life Patients) review service for adults with asthma prescribed fixed-dose combination therapy. Data from 4274 patients were analysed. A major discrepancy between patient-reported and Global Initiative for Asthma defined asthma control was detected; 71.1% of patients who reported ‘well-controlled’ asthma were inaccurate in their perception despite receiving regular maintenance therapy. Significant differences were noted in age, gender, body mass index, education level, medication use, side effects, attitudes to preventer inhaler use, inhaler technique review and respiratory specialist review between patients who accurately reported ‘well-controlled’ asthma and those who did not. Independent risk factors associated with inaccurately reported ‘well-controlled’ asthma were: having taken a maximum of 5–12 puffs or more of reliever inhaler on at least one day within the previous 4 weeks; being female; having seen a respiratory specialist more than a year ago (rather than in the previous year); and having required oral corticosteroids for worsening asthma in the previous year. The study highlighted the significant hidden burden associated with under-recognition of poor asthma control, on the part of the patient and the need for targeted interventions designed to address the continuing discrepancy between perceived and actual disease control.

## Introduction

Asthma is one of the most common chronic conditions worldwide,^1^ affecting ~30 million people in Europe and more than 5 million people in the United Kingdom (UK).^2^ Despite the existence of a global strategy for asthma management,^3^ and availability of national guidelines and effective treatments,^4^ rates of poor asthma control remain high worldwide.^5–20^ Poor asthma control reduces patients’ quality of life and productivity, increases health care utilisation and the risk of exacerbations and mortality.^6,8,21^ Further, poor asthma control poses a greater economic burden on individuals, communities and health care systems than controlled disease.^22,23^

When it comes to disease control, the patient experience of symptoms over time is extremely important. Unfortunately, this is one of the great challenges in asthma management. Many patients who consider their asthma to be ‘well controlled’ often overestimate their actual level of asthma control.^9,19,24,25^ While a discrepancy between patient-perceived and actual disease control is frequently reported, the reasons for and the factors related to this disparity remain unclear. Identifying patients at risk of overestimation of asthma control remains elusive.

The ramifications of overestimating level of disease control on the part of the patient are far-reaching. Many patients who mistakenly consider their asthma to be ‘well controlled’ often accept their symptoms as being part of the ‘norm’ of having asthma and are unlikely to seek asthma-related information, education or care from health care practitioners (HCPs).^26^ Further, since guideline treatment options are based on patient-reported symptoms,^3,4^ appropriate treatment may not be prescribed if a patient fails to recognise and report poorly controlled symptoms to their HCP.^27^ Patients who do not voice their concerns or under-report troublesome symptoms run the risk of under-treatment and the perpetuation of poor asthma control and a potential increased future risk of adverse outcomes.^12^ This under-reporting of symptoms by patients also emphasises the need for more objective measures such as spirometry and symptom questionnaires in clinical practice.

In an attempt to improve rates of well-controlled asthma, a number of studies have identified modifiable and unmodifiable risk factors for poor asthma control across different patient populations.^28–32^ While a broad range of risk factors, both clinical and behavioural, have been identified, assessment of asthma control continues to be based on patient-reported symptoms, hence once again highlighting the importance of the relationship between patient-reported and actual disease control among patients in a real-life clinical setting.^9,33^ To date, little is known about factors associated with overestimation of the level of asthma control. From an HCP perspective, it seems critical to be able to identify patients at risk of overestimating their level of asthma control, and thus inaccurately reporting ‘well-controlled’ asthma. Insight into factors associated with inaccurately reported ‘well-controlled’ asthma may help HCPs’ tailor interventions to patient profiles and help identify effective asthma management strategies for the future. We hypothesised that factors associated with inaccurately reported ‘well-controlled’ asthma among adults with a current physician diagnosis of asthma are patient-related, and some factors are potentially modifiable.

This research aimed to use patient data from the iHARP (initiative Helping Asthma in Real-life Patients) database^34^ to gain deeper insight into the discrepancy between perceived and actual disease control. The specific objectives were to (i) investigate the relationship between patient-reported and actual level of asthma control, (ii) compare the demographic, clinical and attitudinal/behavioural characteristics between patients who believe their asthma is well controlled that accurately report ‘well-controlled’ asthma with those that overestimate their level of asthma control and inaccurately report ‘well-controlled’ asthma and (iii) identify factors associated with inaccurately reported ‘well-controlled’ asthma.

## Results

### Patients

There were 4274 adult patients with asthma-prescribed FDC ICS/LABA therapy in the iHARP database between June 2011 and December 2014. The study cohort included 2574 (60.2%) from the UK, 652 (15.3%) from the Netherlands, 527 (12.3%) from Spain, 403 (9.4%) from Italy, 62 (1.5%) from Sweden, 36 (0.8%) from Norway and 20 (0.5%) from France. The mean (SD) age of patients was 50.9 (14.3) years, 2600 (60.8%) were females, 1416 (33.1%) were obese (body mass index ≥30 kg/m^2^) and 553 (12.9%) were current smokers.

Among the 4274 patients, 1296 (30.3%) patients had controlled asthma, 1912 (44.7%) had partially controlled asthma and 1066 (25.0%) uncontrolled asthma using Global Initiative for Asthma (GINA)-defined criteria (Table 1). When asked about their asthma in the past 4 weeks, 2582 (60.4%) patients believed their asthma was ‘well controlled’. Of this subset of patients, only 745 (28.9%) patients accurately reported ‘well-controlled’ asthma (Group A) and 1837 (71.1%) inaccurately reported ‘well-controlled’ asthma (Group B), that is, they overestimated their level of asthma control (Table 1).

**Table 1:** GINA-defined asthma control overall and by patient-reported level of asthma control.

GINA-defined asthma control^a^	Total, *N* (%) (*N* = 4274)	Patient-reported level of asthma control (*N* = 4274)	
		Well controlled, *n* (%) (*n* = 2582)	Not well controlled, *n* (%) (*n* = 1692)
Controlled	1296 (30.3)	745 (28.9)	551 (32.6)
Partially controlled	1912 (44.7)	1299 (50.3)	613 (36.2)
Uncontrolled	1066 (25.0)	538 (20.8)	528 (31.2)

Note: Data are shown as percentage of patients, *N* = 4274; patients who reported ‘well-controlled’ asthma, *n* = 2582, and those who reported ‘not-well-controlled’ asthma, *n* = 1692

^a^GINA (Global Initiative for Asthma) criteria: daytime symptoms (>2 days/week); need reliever inhaler (>2 days/week); any limitation in daytime activity; any night wakening due to asthma in the past week. The presence of these four criteria determined the asthma control level: none of the above (controlled); 1 or 2 of the above (partially controlled); 3 or 4 of the above (uncontrolled)

Of the 1692 (39.6%) patients who believed their asthma was ‘not well controlled’ in the past 4 weeks, 1141 (67.4%) accurately reported ‘not-well-controlled’ asthma and 551 (32.6%) underestimated their level of asthma control (Table 1). The incidence of accurately reported ‘well controlled’ asthma was significantly lower than that of accurately reported ‘not-well-controlled’ asthma (28.9% vs. 67.4%, *p* = 0.010).

### Characteristics of patients who inaccurately reported ‘well-controlled’ asthma

Patients in Group B who overestimated their asthma control level, that is, inaccurately reported ‘well-controlled’ asthma were significantly more likely to be older, female, obese, or lacking a university degree than those in Group A who accurately estimated ‘well-controlled’ asthma (Table 2). In the week prior to iHARP data collection, 34.2% of Group B patients experienced asthma symptoms for >2 days, 466 (25.4%) indicated that symptoms interfered with their daily activities, 526 (28.6%) had night awakenings due to asthma and 769 (41.9%) used their relievers ≥3 times (Table 2), with 261 (14.2%) indicating they used their relievers ≥10 times. In the 4 weeks prior to data collection, the incidence of taking 5–12 puffs or more of reliever in one day was significantly higher among patients in Group B than those in Group A (13.1% vs. 1.2%, *p* < 0.001) (Table 3). In addition, Group B patients were significantly more likely to report having experienced acute exacerbations requiring short-term courses of oral corticosteroids and asthma-related hospitalisations in the previous year compared with patients in Group A (Table 3).

**Table 2:** Patient demographic characteristics by accurately versus inaccurately reported ‘well-controlled’ asthma.

Characteristic	Patient reported ‘well-controlled’ asthma (*N* = 2582)		*p* Value*
	Group A	Group B	
	Accurately reported (*n* = 745)	Inaccurately reported (*n* = 1837)	
Age, mean (SD)	50.0 (14.4)	51.4 (14.2)	0.02^b^
Age range (years), *n* (%)			
18–29	74 (9.9)	171 (9.3)	0.04^a^
30–49	271 (36.4)	583 (31.7)	
≥50	400 (53.7)	1083 (59.0)	
Gender, *n* (%)			
Female	267 (35.8)	1326 (72.2)	<0.001^a^
Male	478 (64.2)	511 (27.8)	
Body mass category^c^, *n* (%)			
Underweight	12 (1.6)	21 (1.1)	<0.001^a^
Normal	249 (33.4)	563 (30.6)	
Overweight	300 (40.3)	601 (32.7)	
Obese	184 (24.7)	652 (35.5)	
Smoking status, *n* (%)			
Current smoker	72 (9.7)	226 (12.3)	0.08^a^
Ex-smoker	219 (29.4)	566 (30.8)	
Non-smoker	454 (60.9)	1045 (56.9)	
Education^d^, known status, *n* (%)	(*n* = 651)	(*n* = 1500)	
PG or professional degree	30 (4.6)	63 (4.2)	<0.001^a^
University degree^e^	224 (34.4)	508 (33.9)	
Secondary education^e^	278 (42.7)	772 (51.5)	
Primary education^e^	97 (14.9)	131 (8.7)	
None	22 (3.4)	26 (1.7)	

*PG* post graduate, *SD* standard deviation

*p Value of Group A versus Group B

Note: Percentages are column percentages

^a^*χ*^2^ test for independence

^b^Mann–Whitney *U* test

^c^Underweight BMI ≤18.49 kg/m^2^; normal BMI = 18.5–24.99 kg/m^2^; overweight BMI = 25–29.99 kg/m^2^; obese BMI ≥30 kg/m^2^

^d^Estimation of ‘well-controlled’ asthma by education reported as *n* (%) of known status

^e^Completed or some education

**Table 3:** Asthma symptoms and indicators of exacerbations by accurately versus inaccurately reported ‘well-controlled’ asthma.

	Patient reported ‘well-controlled’ asthma (*N* = 2582)		*p* Value*
	Group A	Group B	
	Accurately reported (*n* = 745)	Inaccurately reported (*n* = 1837)	
Asthma symptoms (past 7 days)			
Daytime symptoms, *n* (%)			
None	610 (81.9)	709 (38.6)	<0.001^a^
1–2 days	135 (18.1)	500 (27.2)	
≥ 3 days	0	628 (34.2)	
Activity limitations due to asthma, *n* (%)			
None	745 (100.0)	1371 (74.6)	<0.001^a^
≥1 day	0	466 (25.4)	
Night waking, *n* (%)			
None	745 (100.0)	1311 (71.4)	<0.001^a^
≥1 night	0	526 (28.6)	
Reliever needed for symptoms, *n* (%)			
Not used	607 (81.5)	713 (38.8)	<0.001^a^
1–2 times	136 (18.2)	355 (18.3)	
≥3 times	2 (0.3)	769 (41.9)	
Highest number of puffs of reliever inhaler taken in 1 day^b^, *n* (%)			
0–4	736 (98.8)	1596 (86.9)	<0.001^a^
5–12 or more	9 (1.2)	241 (13.1)	
Acute exacerbations (past 12 months)			
Oral corticosteroid use for worsening asthma, *n* (%)			
None	610 (81.9)	1384 (75.4)	<0.001^a^
1	98 (13.2)	263 (14.3)	
≥2	37 (5.0)	188 (10.3)	
Emergency department visit due to asthma, *n* (%)			
None	694 (93.2)	1680 (91.5)	0.39^a^
1	37 (5.0)	111 (6.0)	
≥2	14 (1.8)	46 (2.5)	
Hospitalisation due to asthma, *n* (%)			
None	730 (98.0)	1751 (95.4)	0.010^a^
1	14 (1.9)	64 (3.5)	
≥2	1 (0.1)	21 (1.1)	

*p Value of Group A versus Group B

^a^Pearson’s *χ*^2^ test for independence

^b^Highest number of puffs of reliever inhaler use, in response to the question: ‘In the past 4 weeks, what was the highest number of puffs in 1 day you took of the reliever inhaler?’ with response options 0, 1–4 puffs and 5+ puffs

Patient-reported inhaler review by an HCP in the previous year, oropharyngeal effects during the inspiration phase of preventer inhaler use and side effects from asthma therapy were more common among patients in Group B than those in Group A (Table 4). Compared to patients in Group A, patients in Group B were significantly more likely to be in agreement that they need to take their inhaler(s) in order for their asthma to be ‘well controlled’ and were significantly more likely to have been reviewed by a respiratory specialist more than a year ago rather than in the previous year (Table 4). The incidence of accurately reported ‘well-controlled’ asthma was significantly higher among patients who had seen a respiratory specialist in the previous year than those who had seen a respiratory physician more than a year ago (31.8% vs. 20.8%, *p* < 0.001).

**Table 4:** Clinical characteristics by accurately versus inaccurately reported ‘well-controlled’, asthma.

	Patient reported ‘well-controlled’ asthma (*N* = 2582)		*p* Value*
	Group A	Group B	
	Accurately reported (*n* = 745)	Inaccurately reported (*n* = 1837)	
Patient-reported rhinitis symptoms, *n* (%)	480 (64.4)	1194 (65.1)	0.75^a^
Patient-reported prior inhaler review by HCP^b^, *n* (%)	348 (46.7)	1027 (56.0)	<0.001^a^
Patient self-assessment of inhaler technique, *n* (%), known	(*n* = 730)	(*n* = 1816)	
Very poor to poor	42 (5.8)	106 (5.8)	0.17^a^
Fair to average	94 (12.9)	287 (15.8)	
Good to excellent	594 (81.4)	1423 (78.4)	
Patient-reported respiratory specialist review	(*n* = 620)	(*n* = 1693)	
Never	242 (39.0)	430 (25.4)	<0.001^a^
In the previous year	107 (17.3)	230 (13.6)	
More than a year ago	271 (43.7)	1033 (61.0)	
Adherence to therapy^c^, *n* (%)	(*n* = 724)	(*n* = 1794)	
Poor	219 (30.2)	569 (31.7)	0.70^a^
Borderline	45 (6.2)	101 (5.6)	
Good	460 (63.5)	1124 (62.7)	
Adherence to therapy in the Netherlands^d^, *n* (%), known	(*n* = 22)	(*n* = 49)	
Poor	7 (31.8)	5 (10.2)	0.06^a^
Borderline	5 (22.7)	9 (18.4)	
Good	10 (45.5)	35 (71.4)	
Patient-reported side effects^e^, *n* (%), known	(*n* = 721)	(*n* = 1793)	
None	507 (70.3)	903 (50.4)	<0.001^a^
1	146 (20.2)	434 (24.2)	
2	52 (7.2)	276 (15.4)	
≥3	16 (2.2)	180 (10.0)	
Patient-reported oropharyngeal effects^g^, *n* (%) known	(*n* = 711)	(*n* = 1766)	
None	452 (63.6)	837 (47.4)	<0.001^a^
1	137 (19.3)	444 (25.1)	
2	88 (12.4)	275 (15.6)	
3	34 (4.8)	210 (11.9)	
Agreement to medication beliefs/perception items, *n* (%) known	(*n* = 721)	(*n* = 1796)	
I need to take my inhaler(s) for my asthma to be well-controlled	460 (63.8)	1273 (70.9)	0.001^a^
I find my inhaler easy to use	649 (90.0)	1616 (90.0)	0.98^a^
Taking regular asthma medication does not worry me	563 (78.1)	1422 (79.2)	0.55^a^

*HCP* health care practitioner

*p Value of Group A versus Group B

^a^ Pearson’s *χ*^2^ test for independence

^b^In the year before an iHARP asthma review

^c^Number (%) calculated as percentage of patients from the UK, Italy, Spain, Sweden, France and Norway reported as *n* (%) of known status

^d^Number (%) calculated as percentage of patients from the Netherlands

^e^Patient-reported side effects from preventer inhaler use, in response to the question: ‘Do you experience any of these side effects from your preventer inhaler?’ with ‘yes’ or ‘no’ responses for the following side effects: continual sore mouth/throat; oral thrush; bruising; hoarse voice; abnormal weight gain and cough. Patients could indicate more than one side effect

^f^Patient-reported oropharyngeal effects during inspiration phase of preventer inhaler use, in response to the question: ‘When you use your preventer inhaler, do you feel a sensation at the back of your throat?; do you sometimes feel a need to cough?; do you feel your medication is deposited at the back of your throat?’ with yes’ or ‘no’ response options. Patients could indicate more than one experience

### Factors associated with inaccurately reported ‘well-controlled’ asthma

Univariate logistic regression models were developed using independent variables as risk factors for inaccurately reported ‘well-controlled’ asthma. The univariate models considered all 12 variables (age group, gender, body mass index, education level, highest number of puffs of reliever taken in one day, oral corticosteroid use for worsening asthma, hospitalisation due to asthma, inhaler review by an HCP, respiratory specialist review, side effects from preventer inhaler use, oropharyngeal effects during the inspiration phase and need to take inhaler(s) for asthma to be well controlled), which were associated with inaccurately reported ‘well-controlled’ asthma. The univariable logistic regression results for the risk of inaccurately reported ‘well-controlled’ asthma are shown in Table 5. There were no correlations between the univariate predictors; therefore, they were subsequently included for analysis in the multivariate logistic regression model. The multivariable logistic regression model was statistically significant (*χ*^2^ *=* 200.37, d.f. = 13, *p* < 0.001), explaining 33.5% (Nagelkerke *R*^2^) of the overall variance. Patients who inaccurately reported ‘well-controlled’ asthma were 14 times more likely to have taken 5–12 puffs or more of reliever in 1 day within the previous 4 weeks (odds ratio (OR) = 14.02, 95% confidence interval (CI):4.19–47.00, *p* ≤ 0.001), nine times more likely to be female (OR = 9.38, 95% CI: 6.23–14.13, *p* < 0.001), nearly nine times more likely to have required oral corticosteroids for worsening asthma in the previous year (OR = 8.80, 95% CI: 2.97–26.06, *p* ≤ 0.001) and nearly four times more likely to have seen a respiratory specialist more than a year ago rather than in the previous year (OR = 3.63, 95% CI: 2.05–6.41, *p* < 0.001) (Table 6). There was a significant interaction between oral corticosteroid use and respiratory specialist review. Among patients who had seen a respiratory specialist in the previous year, those who had not required oral steroids in the previous 12 months were more likely to accurately report ‘well-controlled’ asthma compared to patients who had required oral steroid use. Among patients who had not required oral steroids in the previous 12 months, those who had seen a respiratory specialist in the previous year were more likely to accurately report ‘well-controlled’ asthma compared to those who had seen a respiratory specialist more than a year ago (Table 6).

**Table 5:** Univariable associations between patient characteristics and inaccurately reported ‘well-controlled’ asthma.

	Reference category	Category	Odds ratio (95% CI)	*p* Value
Age group	>50 years	18–50 years	0.80 (0.68–0.95)	0.011
Gender	Male	Female	4.65 (3.88–5.57)	<0.001
Body mass index	Obese	Underweight/normal weight	0.63 (0.51–0.79)	<0.001
Education completed	PG/Professional/University degree	Secondary education	1.24 (1.01–1.51)	0.040
Highest number of puffs of reliever taken in 1 day^a^	5–12 or more	0–4	0.08 (0.04–0.16)	<0.001
Oral corticosteroid use for worsening asthma^b^	≥1	0	0.68 (0.55–0.84)	<0.001
Hospitalisation due to asthma^b^	≥1	0	0.42 (0.24–0.74)	0.002
Inhaler review by HCP^b^	Yes	No	0.69 (0.58-0.82)	<0.001
Respiratory specialist review	More than a year ago	In the previous year	0.56 (0.43–0.74)	<0.001
Side effects from preventer inhaler use	≥1	0	0.43 (0.36–0.52)	<0.001
Oropharyngeal effects during inspiration phase	≥1	0	0.52 (0.43–0.62)	<0.001
Need to take inhaler(s) for asthma to be ‘well-controlled’	Agree	Disagree	0.72 (0.60–0.87)	0.001

*HCP* health care practitioner, *PG* post graduate

^a^In the 4 weeks before an iHARP asthma review consultation

^b^In the year before an iHARP asthma review consultation

**Table 6:** Logistic regression predicting likelihood of inaccurately reporting ‘well-controlled’ asthma.

	Reference category	Category	*B*	Odds ratio (95% CI)	*p* Value
Age group	18–50 years	>50 years	0	1.00 (0.66–1.52)	1.00
Gender	Male	Female	2.24	9.38 (6.23–14.13)	<0.001
Body mass index	Underweight/normal weight	Obese	−0.04	0.96 (0.64–1.45)	0.84
Education level	Secondary education	PG/Professional/University degree	−0.10	0.91 (0.60–1.370	0.64
Highest number of puffs of reliever taken in 1 day^a^	0–4	5–12 or more	2.64	14.02 (4.19–47.00)	<0.001
Oral corticosteroid use for worsening asthma^b^	0	≥1	2.18	8.80 (2.97–26.06)	<0.001
Hospitalisation due to asthma^b^	0	≥1	−0.34	0.71 (0.22–2.31)	0.57
Inhaler review by HCP^b^	No	Yes	−0.11	0.90 (0.59–1.36)	0.61
Respiratory specialist review	In the previous year	More than a year ago	1.29	3.63 (2.05–6.41)	<0.001
Side effects from preventer inhaler use	0	≥1	0.35	1.42 (0.93–2.18)	0.11
Oropharyngeal effects during inspiration phase	0	≥1	0.29	1.33 (0.88–2.01)	0.18
Need to take inhaler(s) for asthma to be ‘well-controlled’	Disagree	Agree	−0.02	0.98 (0.62–1.56)	0.93
Oral corticosteroid use*Respiratory specialist review	0	≥1	−1.97	0.14 (0.04–0.49)	0.002
	In the previous year	More than a year ago			

^a^In the 4 weeks before an iHARP asthma review

^b^In the year before an iHARP asthma review

## Discussion

This research, to our knowledge, is the first to explore the factors that are associated with inaccurately reported ‘well-controlled’ asthma among a large cohort of adult patients who believe their asthma is well controlled and are managed in primary care. In this study, only 30.3% of patients had good asthma symptom control based on GINA-defined criteria despite receiving fixed-dose combination therapy. However, 60.4% of patients considered their asthma to be ‘well controlled’, and of those patients, less than one-third were accurate in reporting well-controlled asthma. Our findings revealed that the incidence of accurately reported ‘well-controlled’ asthma was significantly lower than that of accurately reported ‘not-well-controlled’ asthma. The study provided insight into the ‘silent’ burden of asthma from the patient’s perspective and highlights the potential under-recognition of the increased future risk associated with inaccurately reported well-controlled asthma. Independent risk factors associated with inaccurately reported ‘well-controlled’ asthma were: having taken a maximum of 5–12 puffs or more of reliever inhaler on at least one day within the previous 4 weeks; being female; having seen a respiratory specialist more than a year ago (rather than in the previous year) and having required oral corticosteroids for worsening asthma in the previous year.

This study revealed a major discrepancy between patient-reported and GINA-defined control and a high incidence of inaccurately reported ‘well-controlled’ asthma. While the discrepancy detected in this study is consistent with findings from recent online surveys across Europe,^17,18,24^ Asia,^19^ Australia,^12^ Latin America, Canada and the United States,^13,15,17^ this study is the first to identify factors associated with this discrepancy and highlights the implications for practice. In summary, this study identified that for patients who report good asthma control, 70% will be inaccurate in their perception. From both the patient and HCP perspective, this presents a challenge and questions the patient’s ability to self-assess their asthma and then self-management. From the HCP perspective, it questions the value of asking a patient about their perception of their asthma control or relying on the patient to report uncontrolled disease. It therefore highlights the need to question the patient about their asthma using validated asthma control questionnaires and to be proactive in reviewing the patient’s asthma symptom control and future risk, even when the patient does not recognise this need. This potentially presents a series of HCP-patient relational issues.

Our study provided key insights into the significant increased future risk associated with under-recognition of poor asthma control; that is, patients who overestimate their level of asthma control are more likely to have experienced asthma exacerbations, asthma-related hospitalisations, oropharyngeal effects and/or side effects from asthma therapy, despite having received asthma care and having had their inhaler technique checked in the past. This is somewhat remarkable as these patients report a significant burden of symptoms and medication use: nighttime symptoms, limitations in daily activities, oral corticosteroid use and high dose and/or frequency of short-acting β-agonist (SABA) reliever use, which can increase their future risk of adverse outcomes.^3,19,35^ Importantly, this study revealed that most patients who inaccurately reported ‘well-controlled’ asthma are actually doing what they need to do to manage their asthma in accordance with guideline recommendations;^3,4,36^ that is, they have good medication adherence, hold strong beliefs about the need to take their inhalers for their asthma to be well controlled and are not worried about taking regular asthma medication. These findings suggest that patients are ‘taking control’ of their asthma by doing what they need to do; this flags that they may be interpreting ‘asthma control’ as a personal self-management behaviour (i.e. a verb) rather than a disease outcome (i.e. a noun). This has implications for asthma management practices and patient education, with the need not only to address the perception discrepancy but also for new terminology to promote a shared understanding of asthma symptom control between patients and HCPs.

Identification of independent risk factors for inaccurately reported well-controlled asthma among adults with moderate–severe asthma indicated that these factors are related to patient demographics, rescue medication use and specialist health care under-utilisation; being female, having taken 5–12 puffs or more of reliever inhaler in one day within the previous 4 weeks, having required oral corticosteroids for worsening asthma in the previous year and having seen a respiratory specialist more than a year ago rather than in the previous year. In our study, a significantly higher proportion of females inaccurately reported ‘well-controlled’ asthma compared to males (83.2% vs. 51.7%, *p* < 0.001), which parallels previous findings that females are more likely to have suboptimal levels of asthma control^20^ and more likely to be at risk of making serious inhalation technique errors that are linked to poor asthma control.^37,38^ Our findings add to the body of ‘gender-related’ evidence, and suggest that the self-management tools currently available are not meeting the needs across the genders. There is clearly a need to focus more on giving females the tools that will enable them to become better self-managers of their asthma.

In this study, patients who overestimated their level of asthma control were 14 times more likely to have taken a maximum of 5–12 puffs or more of SABA reliever per day in the previous 4 weeks. This dose of SABA reliever is by far above what is expected and recommended in practice guidelines; that is, 1–2 puffs of SABA as needed for symptom relief during maintenance treatment in asthma.^3,4^ Overuse of SABA may itself potentially contribute to poor asthma control,^39^ increased risk of exacerbations^40,41^ and fatal asthma.^42^ It has been well documented that with chronic or high-dose exposure, β-agonists demonstrate proinflammatory effects, rebound bronchoconstriction and toxicity that can masquerade as worsening asthma.^43^ Further, where overuse is associated with rebound bronchoconstriction, patients will often respond by taking even more SABA medication, thereby entering a vicious cycle of increasing use and progressively worsening symptoms.^43,44^ Our findings indicate a complete misunderstanding of the use of SABA relievers or what we are asking as HCPs, and a lack of awareness of the potential for SABA toxicity to be a significant contributor to poor asthma control. It highlights the deficiency in asking simply on how many times/days per week a patient has used their SABA reliever over the past 4 weeks. Our findings suggest a need for reconsideration of guideline questions that refer to SABA use, and better education of patients, particularly those who have used high doses in the emergency department for acute asthma and continue to use high doses when they have breakthrough symptoms thereafter. In addition, patients who had overestimated their control level were almost nine times more likely to have used oral steroids for worsening symptoms in the previous 12 months reflecting a ‘rescue’ rather than a ‘prevention’ of symptoms approach to asthma management, which is consistent with previous findings,^19^ and not without consequences.^45,46^

Inaccurately reported ‘well-controlled’ asthma was almost four times more likely in patients who had seen a respiratory specialist more than a year ago rather than in the previous year. Our study found that accurately reported ‘well-controlled’ asthma was significantly more likely among patients who had seen a respiratory specialist in the previous year versus more than a year ago, and even more likely among a subset of patients who had not required oral steroids in the previous 12 months, suggesting that specialist referral in the previous year may help improve the discrepancy between patient-perceived and actual disease control. While specialist referral can be effective in improving asthma management, reducing the risk of severe exacerbations and reducing the number of hospitalisations and the risk of future hospitalisations,^47^ there are many barriers that may impede the referral process. These need to be addressed and include overestimation of patients’ actual asthma control by physicians, lack of awareness of guidelines by physicians, lack of adherence to referral guidelines by physicians, communication difficulties between patients and physicians with disparities between patient expectations and experiences with HCPs, financial disincentives for practice stakeholders and low availability of specialists to provide advanced patient care.^9,20,24,48^

Importantly, this study suggests that patient’s perception of their level of asthma control is not linked to preventer medication taking behaviour, as there was no significant difference in adherence rates to fixed-dose combination therapy using the 5-item Medication Adherence Report Scale (MARS) between patients who accurately and inaccurately reported well-controlled asthma. Patient’s perception of their level of asthma control could be influenced by other factors not considered in the analysis, such as ownership of a current written asthma action plan, inhaler technique, knowledge about asthma and trigger factors, asthma-related quality of life, illness perceptions or psychological dysfunction.^9,28,32^ Another possible explanation for our findings, is that the MARS may have poor precision and overestimated adherence rates to preventer medication among this patient population.

The strengths of the study are its large sample size, observational design, real-world evidence, global focus and comprehensiveness, including objective and patient-reported outcomes. This study design enabled the exploration of a heterogeneous, representative population of general practitioner-managed patients with an asthma diagnosis, who were prescribed combination preventer therapy; as this is the most commonly used treatment for asthma, it is likely the findings are generalisable in primary care. There are some potential limitations to this study, which are associated with the cross-sectional study design, which does not allow us to infer cause and effect, and a reliance on patient recall for self-reported symptoms, exacerbations and medication behaviour that may have been under- or over-stated by patients. Additionally, although there were criteria to exclude people with chronic obstructive pulmonary disease (COPD), and given that many people were aged 50 years or more with a past or current history (and a greater risk of COPD), it may have been possible that people with both asthma and COPD were included and could have biased the results. Another limitation of this study relates to the use of an international dataset and possible confounding caused by inter-country differences in how disease outcomes are measured and recorded (e.g. the Netherlands does not use the MARS to assess adherence). A further limitation of this study relates to the high variability in responses caused by recruitment over several countries. Finally, while significant predictors were identified in our multivariate logistic regression model, the overall variance explained by the model (33.5%) was relatively low, suggesting other factors not considered in the current analyses may correlate with inaccurately reported ‘well-controlled’ asthma and reiterating the need for further research in this area. Despite these limitations, this research provided new insight by utilising a structured in-depth asthma review approach and helped in identifying several risk factors associated with inaccurately reported ‘well-controlled’ asthma, which when addressed may help reduce the discrepancy between actual and perceived disease control, improve the incidence of well-controlled asthma and reduce the burden of disease.

The study identified factors associated with patient’s overestimation of their actual level of asthma and highlighted the significant hidden burden associated with under-recognition of poor asthma control, on the part of the patient. It raises the question about the causes for this under-recognition and questions the way in which we ask patients about asthma control. While some of the factors associated with under-recognition may be modifiable, there is an urgent need for targeted interventions that include new strategies, measures and terminology designed to address the continuing discrepancy between perceived and actual disease control. Future qualitative research should explore the causes for under-recognition of poor asthma control as unless we are able to support patients to better align their perception of their asthma with their actual level of asthma control, we will continue to face some fundamental challenges with the management of asthma, and the increased risk of adverse outcomes associated with the poor self-management of this chronic respiratory disease.

## Methods

### Data source

This historical, cross-sectional observational study used anonymised patient data from the iHARP database, an international database comprising anonymised data from practices receiving the iHARP asthma review service. Data were collected prospectively between June 2011 and December 2014 in participating primary care practices in Australia and seven European countries (the United Kingdom (UK), Italy, Spain, France, the Netherlands, Norway and Sweden). The data included patient demographics and results from respiratory reviews led by trained practitioners and asthma questionnaires completed by patients on the day of the iHARP asthma review. For patients assessed within the UK, these data were linked to clinical, diagnostic and prescribing information from electronic medical records.

Each of the participating sites obtained ethics approval for the iHARP asthma review service according to their country-specific requirements. All participants provided informed consent. The study was registered with the European Network of Centres for Pharmacoepidemiology and Pharmacovigilance (ENCEPP) (as ENCePP/SDPP/9651).

### Cohort definition

To be eligible for iHARP review, patients had to have a current diagnosis of asthma, received ≥2 fixed-dose combination inhaled corticosteroid and long-acting β_2_ agonist (FDC ICS/LABA) therapy in the year before the review, and their data had to meet standards of the International Primary Care Respiratory Group,^49^ Quality and Outcomes Framework recommendations^50^ and GINA strategy report at the time of data collection.^51^ Patients were excluded if they had a diagnosis of COPD or any other chronic respiratory disease other than asthma, or if they were receiving long-term systemic asthma treatment (e.g. maintenance oral corticosteroids, theophylline, leukotriene receptor antagonists or anti-IgE therapy), or oral corticosteroids and/or antibiotics for a lower respiratory condition in the 2 weeks before data collection. Our study cohort was restricted to the subset of patients from the database who were at least 18 years of age from participating practices across seven European countries.

### Study data and assessments

Demographics and data from patient-completed asthma questionnaires were obtained from the iHARP database. Asthma control was assessed using the four GINA criteria at the time, which asked patients if they experienced the following during the week preceding the review visit: daytime symptoms (more than twice/week); any night wakening due to asthma; need reliever inhaler (more than twice/week); any limitation in daytime activity. The presence of these four criteria determined the asthma control category as follows: none of the above (controlled); 1 or 2 of the above (partially controlled); 3 or 4 of the above (uncontrolled).^51^

Patient perceptions of their recent asthma control were elicited by asking the patient to respond to the following question: ‘In the past 4 weeks, did you believe that your asthma was ‘well controlled’?’ with ‘yes’ or ‘no’ response options. Patients were categorised in Group A if they indicated they believed that their asthma was ‘well controlled’ AND had controlled asthma as assessed by GINA-defined criteria. Patients were categorised in Group B if they indicated they believed that their asthma was ‘well controlled’ BUT had partially controlled or uncontrolled asthma according to GINA-defined criteria.

Self-reported rhinitis symptoms were assessed with a single question derived from the Allergic Rhinitis and its Impact on Asthma (ARIA) and IPCRG definition of rhinitis;^52,53^ ‘Do you have any of these symptoms: itchy, runny, blocked nose or sneezing when you don’t have a cold?’, with responses ranging from, 0 (‘no’), 1 (‘occasionally and little bother’), 2 (‘occasionally but quite a bother’), 3 (‘most days but little bother’), or 4 (‘most days and a lot of bother’). Responses 1 and 3 were classified under ‘mild’ rhinitis and responses 2 and 4 under ‘moderate–severe’ rhinitis.

All patients were asked if their inhaler technique had been reviewed in the previous year by an HCP, and patients were asked to self-assess their inhaler technique using a Likert scale with scores ranging from 1 (‘I think my inhaler technique is very poor’) to 6 (‘I think my inhaler technique is excellent’). Patients were also asked if they had been reviewed by a specialist respiratory doctor or nurse outside the practice with three response options; ‘in the previous year’, ‘more than a year ago’ or ‘never’.

Beliefs and perceptions about asthma therapy were assessed with three items from the Beliefs about Medicines Questionnaire that were adapted for use in asthma:^54^ ‘I need to take my inhaler(s) for my asthma to be ‘well controlled’; ‘I find my inhalers easy to use’ and ‘Taking regular asthma medication does not worry me,” with ratings on a 6-point Likert scale from 1 (strongly agree) to 6 (strongly disagree).

Self-reported adherence to maintenance therapy was assessed for patients from the UK, Italy, Spain, France, Norway and Sweden using the MARS.^55^ For patients from the Netherlands, adherence to maintenance therapy was assessed with the question, ‘Do you ever forget your prevention inhalation medication?’ Adherence was categorised as poor for responses of ‘very often’ or ‘always’; borderline for responses of ‘now and then’ or ‘regularly’ and good for responses of ‘never’ or ‘rarely’.

Patient-reported experiences during the inspiration phase of preventer inhaler use (i.e. feeling a sensation at the back of the throat, a need to cough and/or of medication being deposited at the back of the throat), side effects from preventer inhaler medications (i.e. continual sore mouth/throat, oral thrush, bruising, hoarse voice, cough and abnormal weight gain) and the highest number of puffs of reliever inhaler taken in 1 day in the previous 4 weeks were recorded during the iHARP review on the patient-completed questionnaire.

Exacerbations were identified by one of the following patient-reported outcomes: hospital admission with breathing or chest problems; emergency department visit related to asthma; or an acute course (5–10 days) of oral corticosteroids for worsening asthma. The number of exacerbations was taken from the 12-month period preceding the iHARP review.

### Data analysis

Analyses were performed using SPSS (IBM® SPSS® Statistics) Version 24. Characteristics of patients who accurately reported ‘well-controlled’ asthma (Group A) were compared to those of patients who inaccurately reported ‘well-controlled’ asthma (Group B). Continuous variables that were normally distributed were compared using Student’s *t* test and the Mann–Whitney *U* test was used for continuous variables that were not normally distributed. Categorical variables were compared using Pearson’s *χ*^2^ test. Univariable logistic regression models, with a dichotomised indicator variable (yes/no) as the dependent variable and each patient characteristic as an explanatory (or predictor) variable, were first used to identify characteristics associated with inaccurately reported ‘well-controlled’ asthma. Indicators of asthma symptoms and asthma control were not tested using univariable logistic regression because they were used to define the dependent variable. Intercorrelations among the predictor variables were then tested using Tolerance values, and those with high intercorrelations were not included in subsequent analyses. Demographic and clinical characteristics associated with inaccurately reported ‘well-controlled’ asthma in the univariable model (*p* < 0.05) were then entered into a multivariable regression model to produce a final list of non-collinear independently associated variables. Interactions between different variables were tested, and those that did not improve the model were not included. The goodness of fit of the logistic regression model was confirmed by the Hosmer and Lemeshow test. A two-tailed significance level of 0.05 was used for all statistical procedures.

## Supplementary information

Authors email addresses

## Data Availability

The data that support the findings of this study are available from the corresponding upon reasonable request.
