# Supplementary material for: A multinational observational study identifying primary care patients at risk of overestimation of asthma control
Source: NPJ Prim Care Respir Med. 2019 Dec 5;29:43. doi: 10.1038/s41533-019-0156-4 (PMC6895161; doi:10.1038/s41533-019-0156-4)
Supplement: Supplementary file 1 — Authors email addresses [file 41533_2019_156_MOESM1_ESM.docx]

**Authors’ email addresses:**

Vicky Kritikos: vicky.kritikos@sydney.edu.au

David Price: [dprice@opri.sg](mailto:dprice@opri.sg)

Alberto Papi: [ppa@unife.it](mailto:ppa@unife.it)

Antonio Infantino: antonio.infantino@gmail.com

Bjorn Ställberg: [b.stallberg@telia.com](mailto:b.stallberg@telia.com)

Dermot Ryan: [dermotryan@doctors.org.uk](mailto:dermotryan@doctors.org.uk)

Federico Lavorini: [federico.lavorini@unifi.it](mailto:federico.lavorini@unifi.it)

Henry Chrystyn: [h.chrystyn@gmail.com](mailto:h.chrystyn@gmail.vom)

John Haughney: j.haughney@abdn.ac.uk

Karin Lisspers: karin.lisspers@ltdalarna.se

Kevin Gruffydd-Jones: gruffbox@btinternet.com

Miguel Román Rodríguez: miguelroman@ibsalut.caib.es

Svein Høegh Henrichsen: svein.hoegh.henrichsen@helsedir.no

Thys van der Molen: thysvandermolen@gmail.com

Victoria Carter: victoria@opri.sg

Sinthia Bosnic-Anticevich: sinthia.bosnic-anticevich@sydney.edu.au
